# Optimizing hepatitis B vaccination in chronic kidney disease: a comprehensive scoping review of strategies across CKD stages, dialysis, and transplant populations

**DOI:** 10.1080/0886022X.2025.2541873

**Published:** 2025-08-07

**Authors:** Wiwat Chancharoenthana, Opas Traitanon, Asada Leelahavanichkul, Claudio Ronco

**Affiliations:** a Department of Clinical Tropical Medicine, Faculty of Tropical Medicine, Mahidol University, Bangkok, Thailand; b Tropical Immunology and Translational Research Unit (TITRU), Department of Clinical Tropical Medicine, Faculty of Tropical Medicine, Mahidol University, Bangkok, Thailand; c Division of Nephrology, Department of Medicine, Faculty of Medicine, Thammasat University, Pathumthani, Thailand; d Department of Microbiology, Faculty of Medicine, Chulalongkorn University, Bangkok, Thailand; e Center of Excellence in Translational Research in Inflammation and Immunology (CETRII), Department of Microbiology, Faculty of Medicine, Chulalongkorn University, Bangkok, Thailand; f International Renal Research Institute of Vicenza (IRRIV), Vicenza, Italy; g Department of Nephrology, Dialysis and Transplantation, San Bortolo Hospital, Vicenza, Italy; h Department of Medicine (DIMED), Università Degli Studi di Padova, Padua, Italy

**Keywords:** Hepatitis B vaccination, chronic kidney disease, adjuvanted vaccines, seroprotection, immunogenicity, hemodialysis

## Abstract

This scoping review aims to comprehensively map and critically analyze existing evidence regarding the optimal HBV vaccination strategies—including vaccine types, schedules, immunogenicity, duration of protection, factors influencing seroresponses, and safety—primarily in adults with pre-dialysis and dialysis-dependent chronic kidney disease (CKD) populations. Patients with CKD are at increased risk of hepatitis B virus (HBV) infection due to the immune dysfunction with frequent blood exposure during invasive procedures, including hemodialysis. Although HBV vaccination is crucial for CKD patients due to the high transmission efficacy of HBV, enhanced regimens or adjuvants are often required because of CKD-induced immune dysfunction. This scoping review followed Joanna Briggs Institute guidelines and the PRISMA-ScR checklist through searching on PubMed, EMBASE, and SCOPUS for English-language studies until August 2024, demonstrated 329 unique records, and 17 studies were included. Accordingly, 4 times of either double-dose simple recombinant vaccine (Engerix-B 40 mcg) or adjuvanted vaccines (e.g., Fendrix^®^, Heplisav-B^®^ 20 mcg) demonstrated higher seroprotection rates than the standard conventional schedule (3 times of Engerix-B 20 mcg). Vaccination at pre-dialysis stages (eGFR > 15 mL/min/1.73 m^2^) yielded higher short-term seroprotection (SPR: 63–100% at 1-2 months after the last dose), than in dialysis patients (SPR: 50–89.3% at 1–2 months after the last dose) and the antibody titers were more prominent with adjuvant vaccines than with the nonadjuvant one. In conclusion, the higher doses of vaccination are required in adult patients with CKD; however, data on transplant recipients and pediatric patients are very limited. Large-scale, high-quality randomized controlled trials with long-term immunity monitoring are needed.

## Introduction

Patients with chronic kidney disease (CKD) are at a higher risk of hepatitis B virus (HBV) infection compared to healthy individuals. In the general population, the World Health Organization (WHO) estimated the global prevalence of chronic hepatitis B at approximately 3.3% (254 million people) in 2022, with an incidence of new HBV infections at 1.23 million [1]. In contrast, the prevalence of HBV infection in hemodialysis patients ranges from 4% to 10%, depending on geographic region and dialysis status [2]. This elevated risk in the CKD population, particularly those on hemodialysis, underscores the critical need for effective vaccination strategies.

The CKD-induced immune dysfunction, along with the increased exposure to blood products and invasive procedures during dialysis, makes these patients more susceptible to HBV infection [3,4]. Then, vaccination against hepatitis B is an important preventive strategy for CKD patients, providing protection against this devastating viral infection. Furthermore, studies have found a correlation between positive response to hepatitis B vaccination and reduced risk of all-cause mortality and cardiovascular events [5]. Perhaps the good responses to HBV vaccination are associated with the better immune status in patients with CKD, which also contributes to better overall health outcomes for the patients. For instance, a stronger immune response to a vaccine indicates immune competence, enabling better defense against infections and inflammatory control that reduces CKD-related complications, thereby lowering overall mortality risks [6]. Also, the HBV prevention infection mitigates risks of liver-related morbidity and mortality, which can contribute to overall mortality in this vulnerable population. The linkage between immune responses against HBV and the general healthiness of patients might be useful for the determination of patient’s conditions and CKD complication monitoring.

The reduced immune response in CKD patients presents a significant challenge, often requiring higher vaccine doses, different vaccination schedules, or the use of adjuvants to improve immunogenicity [3,7]. Traditional recombinant HBV vaccines paired with alum adjuvants (such as Engerix-B^®^, Recombivax^®^, Euvax-B^®^) have been shown to be safe with effective responses in healthy individuals [8,9]. However, more potent adjuvants, like ASO4 (Fendrix^®^) and CPG1018 (Heplisav-B^®^), are needed to enhance immunogenicity, especially in patients with CKD [10]. Despite these available strategies, significant variations are demonstrated across clinical practices regarding vaccine type, dosing schedules, and follow-up protocols.

This scoping review aims to comprehensively map and critically analyze evidence on optimal HBV vaccination strategies for CKD patients across different stages, primarily focusing on pre-dialysis and dialysis-dependent adults. The key aspects, such as vaccine immunogenicity, duration of protection, factors influencing seroresponses, and the safety of various vaccination approaches, were incorporated. Notably, the data on HBV vaccination in post-transplant populations and pediatric CKD was very limited and was not included in this review. By highlighting the strengths and limitations of current practices and identifying knowledge gaps, this review seeks to inform future clinical guidelines and research priorities, ultimately enhancing patient outcomes.

## Methods

**Figure 1.**
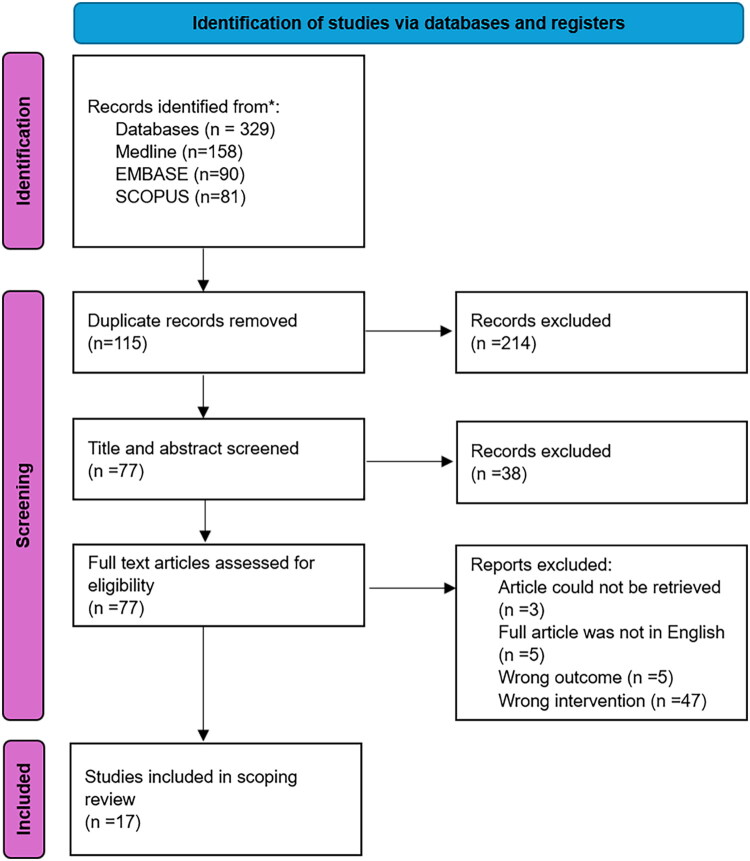
PRISMA flow chart.

This scoping review was conducted in alignment with the Joanna Briggs Institute guidelines on scoping reviews [11] and the PRISMA extension for Scoping Reviews (PRISMA-ScR) checklist [12] (Figure 1 and Appendix A1).

### Search strategy

A comprehensive search strategy was developed and executed across PubMed, EMBASE, and SCOPUS from their inception to August 2024. The search strategy combined keywords and MeSH/Emtree terms related to ‘Hepatitis B’, ‘Hepatitis B Vaccines’ (including specific vaccine names like ‘Engerix-B’, ‘Fendrix’, ‘Heplisav-B’), and ‘Chronic Kidney Disease’ (including terms like ‘renal insufficiency’, ‘end-stage renal disease’, ‘hemodialysis’, ‘peritoneal dialysis’, ‘kidney transplant recipients’). Boolean operators (AND, OR) were used to combine search terms. No language restrictions were applied during the search phase, but only English language publications were included for final analysis. The detailed search strategy for each database, including all search terms, combinations, and any filters applied (e.g., publication types, human studies), is provided in Appendix A2. Additional searches for peer-reviewed and non-peer-reviewed material were conducted by examining bibliographies and conducting additional internet searches using Google Scholar. Titles, abstracts, and full texts were carefully examined based on the specified criteria for inclusion and exclusion. Only the articles that met the criteria were chosen for further analysis.

### Inclusion and exclusion criteria

Studies were screened by title, abstract, and full text against detailed inclusion and exclusion criteria, as presented in Table 1.

**Table 1.** Detailed inclusion and exclusion criteria for scoping review.

Criteria Category	Inclusion Criteria	Exclusion Criteria
Publication type	Peer-reviewed original research articles (e.g. randomized controlled trials, cohort studies, case-control studies, cross-sectional studies providing primary data), pre-prints reporting primary data.	Non-empirical works (e.g. reviews without new data synthesis, editorials, letters without original data, case reports on single patients unless providing unique data on a specific strategy not found elsewhere).
Population	Adult and pediatric patients with any stage of CKD (including pre-dialysis eGFR <60 mL/min/1.73m², dialysis-dependent [hemodialysis, peritoneal dialysis], and kidney transplant recipients).	Studies where CKD patient data cannot be disaggregated from a larger mixed population. Animal studies.
Intervention	Any HBV vaccination strategy (e.g., type of vaccine, dosage, schedule, route of administration).	Studies not focused on HBV vaccination.
Comparator	Other HBV vaccination strategies, placebo, or no vaccination (if providing relevant immunogenicity data for a specific strategy).	Not applicable if descriptive study of a single strategy providing primary immunogenicity data.
Outcomes	Must report at least one of the following: seroprotection rate (defined as anti-HBs ≥10 mIU/mL), seroconversion rate, geometric mean titers (GMT) of anti-HBs, duration of seroprotection, or safety/adverse event data related to HBV vaccination.	Studies not reporting immunogenicity or safety outcomes specific to HBV vaccination in CKD patients.
Language	English language full-text publication.	Non-English full-text publications.
Minimum follow-up	No minimum follow-up required for inclusion (to map both short-term [e.g., 1–2 months postvaccination] and long-term data); studies were grouped by follow-up duration in the synthesis.	Not applicable (no exclusions based on follow-up length).
Study quality thresholds	No quality thresholds applied for exclusion (per scoping review methodology); however, risk of bias was assessed for all included studies using RoB 2 for RCTs and ROBINS-I for non-randomized studies to contextualize findings.	Not applicable (no exclusions based on quality).

CKD: chronic kidney disease; eGFR: estimated glomerular filtration rate; HBV: hepatitis B virus; anti-HBs: hepatitis B surface antibody.

### Data extraction

Data were extracted by two independent reviewers (WC and AL) using a standardized data extraction form, with discrepancies resolved by consensus or a third reviewer. Extracted information included: study identifiers (author, year), study design, country, population characteristics (CKD stage, dialysis modality, transplant status, age, sample size, key comorbidities), details of the HBV vaccination strategy (vaccine type, brand name, adjuvant, dose, number of doses, schedule, route of administration), reported immunogenicity outcomes (seroprotection rates, seroconversion rates, anti-HBs titers at specified time points post-vaccination), duration of protection data, safety data (types and frequency of adverse events), and reported statistical measures (e.g. p-values, confidence intervals [CIs]). Where available, 95% CIs for seroprotection rates and p-values for comparisons were extracted. This standardized approach to data extraction is crucial for ensuring consistency in the data collected, which underpins the reliability of the subsequent synthesis and presentation of results.

### Assessment of methodological quality/risk of bias in included studies

The methodological quality and risk of bias of included studies were independently assessed by two reviewers. For randomized controlled trials (RCTs), the Cochrane Risk of Bias 2 (RoB 2) tool [13] was utilized, evaluating domains such as bias arising from the randomization process, deviations from intended interventions, missing outcome data, measurement of the outcome, and selection of the reported result. For non-randomized studies of interventions (e.g. prospective cohort studies), the Risk Of Bias In Non-randomized Studies - of Interventions (ROBINS-I) tool [14] was applied, covering domains like confounding, selection of participants, classification of interventions, deviations from intended interventions, missing data, measurement of outcomes, and selection of the reported result. Disagreements were resolved through discussion or consultation with a third reviewer. The results of these assessments were not used to exclude studies but to provide context to the interpretation of the findings and are summarized in Appendix A3. The inclusion of this formal risk of bias assessment addresses a critical component of evidence synthesis, allowing for a more nuanced understanding of the reliability of the findings from individual studies and the overall strength of the evidence base.

### Data synthesis

Due to the anticipated heterogeneity in study designs, populations, interventions, and outcome reporting, a formal meta-analysis was not planned. Instead, a structured narrative synthesis approach was adopted, guided by the principles outlined in the Synthesis Without Meta-analysis (SWiM) reporting guideline [15]. This involved: (1) grouping studies based on CKD stage (pre-dialysis, dialysis, transplant), pediatric status, vaccine type (conventional vs. adjuvanted), and vaccination schedule (e.g. standard vs. accelerated, dose levels); (2) standardizing the presentation of the primary outcome (seroprotection rate at 1–2 months postvaccination completion, defined as anti-HBs ≥10 mIU/mL); (3) summarizing findings within these groups, focusing on patterns of seroprotection, duration of immunity, influencing factors, and safety; and (4) exploring heterogeneity qualitatively by comparing findings across these subgroups. Findings are presented in textual summaries and supported by comprehensive tables. Adopting the SWiM framework provides a transparent and methodologically sound approach for synthesizing diverse evidence when meta-analysis is not feasible, thereby strengthening the review’s conclusions.

## Results

### Search results

From 329 unique records, 77 underwent full-text review, and 17 met inclusion criteria **(**Figure 1**).** The list of included studies is provided in Tables 2 and 3. A summary of the risk of bias assessment for these studies is presented in Appendix A3.

**Table 2.** Summary of studies on hepatitis B vaccination in pre-dialysis CKD patients.

Author (Year) (Reference)	Study Design, Site	Population	Vaccine & Schedule	Sample (N, Vaccine/Control or Total)	SPR (%) at X Month(s) After the Last Dose (95% CI; p-value vs comparator if applicable)	Duration of Response (e.g., % SPR at 1 yr)
McNulty CA, et al. (2005) [54]	RCT, UK	Pre-dialysis, Cr 3.6-10.8 mg/dL	Engerix-B^®^ 20 mcg at 0,1,6 mo	*N* = 60	57 (at 1 mo)	Not reported
			Engerix-B^®^ 40 mcg at 0,1,6 mo	*N* = 61	67 (at 1 mo)	Not reported
Garcia-Agudo R, et al. (2012) [36]	Prospective cohort, Spain	CKD for transplant, eGFR ≈28 mL/min	Engerix-B^®^ 40 mcg 0,1,2,6 (up to 2 cycles) then Fendrix^®^ 20 mcg 0,1,2,6 mo	*N* = 155 total	93.8 (post Engerix^®^); 97.5 (post Fendrix^®^) (at 1 mo)	Not reported
Janssen RS, et al. (2013) [19]	Multicenter RCT, USA, Germany, Canada	CKD, eGFR ≈16-45 mL/min	Heplisav-B^®^ 20 mcg at 0,1,6 mo	*N* = 261 (Heplisav)	89 (at 1 mo after the last dose, i.e. month 7)	78% at 1 yr (Heplisav-B^®^)
			Engerix-B^®^ 40 mcg at 0,1,2,6 mo	*N* = 260 (Engerix)	81 (at 1 mo after the last dose, i.e. month 7)	Not reported
Janssen M, et al. (2015) [22]	RCT, observer-blinded, USA	Diabetic CKD, eGFR ≈16-45 mL/min	Heplisav-B^®^ 20 mcg at 0,1,6 mo	*N* = 165 (Heplisav)	89 (at 1 mo after the last dose)	Not reported
			Engerix-B^®^ 40 mcg at 0,1,2,6 mo	*N* = 163 (Engerix)	76 (at 1 mo after the last dose)	Not reported
Krairittichai U, et al. (2017) [18]	RCT, Thailand	CKD, eGFR ≈46 mL/min	Engerix-B^®^ 20 mcg at 0,1,2,6 mo	*N* = 20	95 (at 1 mo after the last dose)	Not reported
			Engerix-B^®^ 40 mcg at 0,1,2,6 mo	*N* = 19	100 (at 1 mo after the last dose)	Not reported
Fabrizi F, et al. (2020) [21]	Prospective cohort, Italy	CKD, eGFR ≈19 mL/min	Fendrix^®^ 20 mcg at 0,1,2,3 mo	*N* = 107	95 (at 4th month, i.e. 1 mo after the last dose)	Not reported
Hettenbaugh J, et al. (2021) [55]	Prospective cohort, USA	CKD, eGFR ≈20 mL/min	Engerix-B^®^ 40 mcg at 0,1,6 mo	*N* = 107	43^a^(timing not specified, likely 1 mo after the last dose)	Not reported
Feng Y, et al. (2021) [56]	RCT, China	Pre-dialysis (40% eGFR >90)	Engerix-B^®^ 20 mcg at 0,1,6 mo; or 0,1,2,6 mo; or 60 mcg at 0,1,2,6 mo	N not specified per arm	66-68 (across groups, at 1 mo after the last dose)	Not reported
Kittrakulrat J, et al. (2024) [20]	RCT, Thailand	CKD, eGFR <30 mL/min	Engerix-B^®^ 40 mcg at 0,1,4,8 wks	*N* = 66	83 (95% CI: 74.5-89.2%) (at 12 wks)	Not reported
			Engerix-B^®^ 40 mcg at 0,1,2,6 mo	*N* = 67	63.2 (95% CI: 52.4-72.9%) (at 12 wks, *p* = 0.006 vs accelerated at 12 wks)	Not reported

SPR: Seroprotection rate, defined as anti-HBs ≥ 10 mIU/mL, unless otherwise specified; CI: Confidence Interval; eGFR: estimated glomerular filtration rate in mL/min/1.73m^2^; Cr: Creatinine.

^a^Using anti-HBs >12 mIU/mL as cutoff for seroconversion.

Note: SPR timing reflects one-month after the last dose unless specified otherwise.

**Table 3.** Summary of studies on hepatitis B vaccination in dialysis patients.

Author (Year) [Reference]	Study Design, Site	Population	Vaccine & Schedule	Sample Size (N, Vaccine/Control or Total)	SPR (%) at X Month(s) After the Last Dose (95% CI; p-value vs comparator if applicable)	Duration of Response (e.g., % SPR at 1 yr)
Tong N, et al. (2005) [30]	Multicenter RCT, Spain, Czech Rep, Malaysia	HD (53%) & CKD eGFR <30 (47%)	Engerix-B^®^ 40 mcg at 0,1,2,6 mo	*N* = 81 (Engerix)	84 (at 1 mo after the last dose)	Not reported
			Fendrix^®^ 20 mcg at 0,1,2,6 mo	*N* = 84 (Fendrix)	90 (at 1 mo after the last dose)	Not reported
Lacson E, et al. (2005) [26]	Retrospective, USA	Dialysis (HD 93.5%, PD 6.5%)	Engerix-B^®^ 40 mcg at 0,1,2,6 mo	N not specified per arm	58 (timing post-vaccination not specified)	Not reported
			Recombivax^®^ 40 mcg at 0,1,6 mo	(Total *N* = 14,546)	40 (timing post-vaccination not specified)	Not reported
Polito P, et al. (2011) [34]	RCT, Italy	HD patients	Fendrix^®^ 20 mcg at 0,1,2,6 mo (IM)	*N* = 46	48 (at 1 mo after the last dose)	Not reported
Chow KM, et al. (2012) [57]	Multicenter RCT, Hong Kong	Peritoneal dialysis patients	Engerix-B^®^ 80 mcg at 0,1,6 mo	*N* = 55	62 (at 1 mo after the last dose)	Not reported
			Engerix-B^®^ 40 mcg at 0,1,6 mo	*N* = 54	78 (at 1 mo after the last dose)	Not reported
Fabrizi F, et al. (2015) [53]	Prospective cohort, Italy	Dialysis (HD 91%)	Fendrix^®^ 20 mcg at 0,1,2,3 mo	*N* = 102	84 (at 4th month, i.e. 1 mo after the last dose)	Not reported
Awad AM, et al. (2021) [27]	Open-label single-arm, USA	HD patients	Heplisav-B^®^ 20 mcg at 0,1,2,4 mo	*N* = 119	89.3 (95% CI: 82.3-94.2%) (at 5th month, i.e. 1 mo after the last dose)	96% at 1 yr
Manley HJ, et al. (2023) [28]	Retrospective cohort, USA	HD patients	Heplisav-B^®^ 20 mcg at 0,1 mo	*N* = 1672 (Heplisav)	50-60 (timing post-vaccination not specified)	Not reported
			Engerix-B^®^ 40 mcg at 0,1,2,6 mo	*N* = 2176 (Engerix)	50-60 (timing post-vaccination not specified)	Not reported
Walsh C, et al. (2023) [29]	Case series, USA	HD patients, all Hispanic	Heplisav-B^®^ 20 mcg at 0,1,6 mo	*N* = 32	83 (timing post-vaccination not specified)	Not reported
Kamath N, et al. (2019) [44]	Prospective study, UAE	Pediatric CKD stage II-IV and dialysis	Standard three-dose HBV vaccine (not specified)	*N* = 36	72 (at 1-2 months after the last dose); 60 after second series for non-responders	60% at 1 year

SPR: Seroprotection rate, defined as anti-HBs ≥ 10 mIU/mL; CI: Confidence Interval; HD: Hemodialysis; PD: Peritoneal Dialysis; eGFR: estimated glomerular filtration rate in mL/min/1.73m^2^; IM: Intramuscular.

Note: For studies with mixed populations, SPR is reported for the combined group unless specified. Where SPR timing deviates, it reflects one-month after the last dose unless specified otherwise.

### Study overview

The 17 included studies comprised nine randomized controlled trials and 8 observational studies (prospective cohort, retrospective, or single-arm studies). These studies investigated HBV vaccination in diverse CKD populations: 7 studies focused primarily on pre-dialysis patients **(**Table 2**)**, and 8 on dialysis-dependent patients **(**Table 3**)**, while specific data on primary vaccination series initiated in kidney transplant recipients and pediatric CKD patients were more limited within these broader studies or addressed specific aspects like duration of immunity from prior vaccination. A range of vaccine types were evaluated, including conventional alum-adjuvanted vaccines (e.g. Engerix-B^®^, Recombivax^®^) and newer vaccines with potent adjuvants such as AS04 (Fendrix^®^) or CpG 1018 (Heplisav-B^®^). The specific vaccines included across the studies were Engerix-B^®^ (most commonly evaluated, in 10 studies), Recombivax^®^ (in 1 study), Fendrix^®^ (in 4 studies), and Heplisav-B^®^ (in 5 studies), often in comparative designs or single-arm evaluations. Various dosages and schedules were reported, reflecting the ongoing efforts to optimize HBV immunogenicity in this hard-to-vaccinate population. Conventional vaccines like Engerix-B^®^ and Recombivax^®^ are widely used in standard clinical practice globally, typically with enhanced regimens (e.g. higher doses or additional shots) for CKD patients due to their established safety and availability. Adjuvanted vaccines such as Fendrix^®^ and Heplisav-B^®^ are increasingly incorporated into standard practice where available, as they offer improved immunogenicity; however, regional differences exist, with Fendrix^®^ licensed primarily in Europe for CKD populations (not available in the US), while Heplisav-B^®^ is approved and gaining use in the US (and more recently in the EU), influencing local guidelines and adoption based on regulatory approvals and healthcare systems.

### Immunogenicity and safety findings

#### Pre-dialysis CKD patients

For patients with earlier stages of CKD, it is recommended that they receive HBV vaccination before starting dialysis [16,17]. This early intervention helps ensure a better immune response and protection against HBV infection.

For individuals in the early CKD group (eGFR > 45 mL/min/1.73 m^2^), there is no available data from the included studies to definitively determine whether a double-dose regimen is necessary. One study showed that the standard dose regimen achieved almost the same seroresponse rate, but the study had a small sample size (*N* = 39) and was underpowered for a conclusion. Specifically, Krairittichai et al. [18] reported seroprotection rates of 95% (19/20) with Engerix-B^®^ 20 mcg (0, 1, 2, and 6 months) versus 100% with Engerix-B^®^ 40 mcg (0, 1, 2, and 6 months) in CKD patients with a mean eGFR of approximately 46 mL/min/1.73 m^2^ (*N* = 39 total), but the difference was not statistically significant (*p* = 0.323; no CI reported) [18]. The study concluded that seroconversion rates were not significantly different after completion of vaccination between the single dose and double dose, but the double dose maintained higher persistent anti-HBV at 6 months post-completion (*p* = 0.040) [18].

In pre-dialysis patients with more advanced CKD (stage 3b-5; eGFR < 45 mL/min/1.73 m^2^), the literature supports a schedule of four shots of the double-dose conventional recombinant hepatitis B vaccine, HepB-Eng (Engerix-B^®^). Likewise, vaccination before dialysis (eGFR > 15 mL/min/1.73 m^2^) optimizes immune response [16,17]. Four double-dose Engerix-B^®^ (40 mcg) shots at 0, 1, 2, and 6 months achieved short-term seroprotection rates of 63-100% at 1-month after the last dose **(**Table 2**)**. For example, Janssen et al. [19] in a multicenter RCT (*N* = 521, mean eGFR ∼16-45 mL/min/1.73 m^2^) found an 81% (no CI reported) seroprotection rate (SPR) at 1 month after the last dose with four 40 mcg doses of Engerix-B^®^ [19], concluding that the standard regimen provides good seroprotection but is inferior to the investigational vaccine in terms of the antibody titers.

An accelerated schedule (0, 1, 4, and 8 weeks) of Engerix-B^®^ 40 mcg yielded 83% seroprotection at 12 weeks versus 63% with the standard schedule (0, 1, 2, and 6 months) when also measured at 12 weeks (i.e. prior to completion of the full standard schedule) in patients with eGFR <3 0 mL/min/1.73 m^2^ (*N* = 133 total; (83% [95% CI: 74.5-89.2%] vs. 63.2% [95% CI: 52.4-72.9%], *p* = 0.006), indicating that the accelerated schedule achieved seroprotection significantly earlier, although the final seroprotection after completion of the standard schedule might differ [20]. This suggests that the accelerated schedule offers superior early protection. Notably, the 12-week assessment was conducted 4 weeks after the third dose of the standard schedule (administered at 0, 1, and 2 months), rendering the standard regimen incomplete at this interim timepoint, as it requires a fourth dose at 6 months for the full completion [20].

Adjuvanted vaccines have shown seroprotection rates of 89-95% (no CI reported) in this group, compared to 63-100% (with varying CIs as above) for enhanced conventional regimens (Engerix-B^®^ 40 mcg 4 times injection). HepB-AS04 (Fendrix^®^), administered as four 20 mcg doses, achieved a 95% (97/102, no CI reported) SPR at 1 month after the last dose (at the 4^th^ month) in CKD patients with a mean eGFR of approximately 19 mL/min/1.73 m^2^ (*N* = 107) [21], concluding that HBV-AS04 is highly immunogenic and safe in pre-dialysis CKD. HepB-CpG (Heplisav-B^®^), given as three 20 mcg doses at 0, 1, and 6 months, reported an 89% (no CI reported) SPR at 1 month after the last dose in CKD patients (mean eGFR ∼16-45 mL/min/1.73 m^2^, *N* = 521) [19], concluding it is superior to Engerix in higher titers (73.6% vs 63.2% for the antibody titers higher than 100 mIU/mL, *p* < 0.05), and a similar 89% (no CI reported) in diabetic CKD patients (mean eGFR ∼16-45 mL/min/1.73 m^2^, *N* = 328) with a 0, 1, and 6 months schedule [22], concluding Heplisav-B induced a higher SPR than Engerix-B in diabetic CKD patients. These findings align with KDIGO guidelines recommending HBV vaccination pre-dialysis [17]. The superior seroprotection observed with adjuvanted vaccines like HepB-AS04 (Fendrix^®^) and HepB-CpG (Heplisav-B^®^) in pre-dialysis patients suggests these could be considered as first-line options or alternatives to high-dose conventional vaccines, potentially leading to revisions in future vaccination guidelines for this population, pending further comparative effectiveness and cost-effectiveness studies [10,23,24].

#### Dialysis patients

Seroresponse rates in hemodialysis patients vary widely. Standard HBV vaccine schedules in hemodialysis patients often involve higher antigen doses or more frequent injections compared to the general population [5,7]. For instance, a common regimen for Engerix-B^®^ is four double doses (40 mcg each) administered at 0, 1, 2, and 6 months [25,26]. Heplisav-B^®^ (HepB-CpG), an adjuvanted vaccine containing the CpG 1018 oligonucleotide, is typically administered as two 20 mcg doses one month apart in the general adult population, but studies in hemodialysis patients have evaluated schedules with 2 to 4 doses [27–29]. Fendrix^®^ (HepB-AS04), which contains the AS04 adjuvant system combining monophosphoryl lipid A (MPL) and aluminum phosphate, is licensed in Europe specifically for CKD patients (including those on dialysis) with a four-dose schedule (20 mcg at 0, 1, 2, and 6 months) [30]. Regional guidelines, such as those from the US CDC/ACIP and European bodies, reflect these intensified approaches, though specific vaccine availability (e.g., Fendrix^®^ not being available in the US, Heplisav-B^®^ gaining traction) influences local practices [31,32].

In hemodialysis patients, Heplisav-B^®^ (20 mcg, 2–4 doses) achieved short-term seroprotection rates ranging from 50% to 89.3% at 1–2 months after the last dose [27–29]. For instance, Awad et al. [27], in an open-label, single-arm study (*N* = 119), reported a seroprotection rate of 89.3% (95% CI: 82.3–94.2%) at the 5th month (one month after the last dose) with a four-dose Heplisav-B^®^ schedule (0, 1, 2, and 4 months) [27], concluding high immunogenicity and safety with 4 doses in HD patients. This outperformed historical rates often seen with HepB-Eng (Engerix-B^®^) (40 mcg, four doses), which one study cited as achieving 58% (N not specified per arm; no CI or p-value reported) [26]. However, a large real-world retrospective cohort study by Manley et al. [28] (*N* = 3,848 dialysis patients) found that two doses of 20 mcg Heplisav-B^®^ (0, 1 month) resulted in seroprotection rates of 50.1% (no CI reported), which was lower than the 62.9% (no CI reported) achieved with four doses of 40 mcg HepB-Eng (Engerix-B^®^) in the same study population (*p* < 0.0001) [28], but up to 4 doses of Heplisav-B yielded 82.0% vs 62.9% for Engerix-B (*p* < 0.0001), concluding that optimal Heplisav-B dosing may require more than 2 doses for better seroprotection. This discrepancy between RCT efficacy and real-world effectiveness highlights the importance of considering different study designs and patient populations when evaluating vaccine performance.

HepB-AS04 (Fendrix^®^) (20 mcg, four doses at 0, 1, 2, and 6 months) reached a 90% (no CI reported) SPR at 1 month after the last dose in a multicenter RCT that included 53% hemodialysis patients (*N* = 165 total, with a subgroup of HD patients) [30], superior to the 84% (no CI reported) achieved with four double doses (40 mcg) of Engerix-B^®^ in the same study (p-value not reported for direct comparison) [30], concluding HB-AS04 provides higher and more durable seroprotection. Conversely, some real-world data indicate that four doses of 40 mcg HepB-Eng (Engerix-B^®^) did not provide adequate seroprotection, with rates reported to be as low as 50-60% [28].

Intradermal (ID) administration of HBV vaccines has been explored as a strategy to improve response rates, particularly in the non-responders using the intramuscular (IM) vaccination regimens [33]. A systematic review focusing on hemodialysis patients indicated that intradermal administration could lead to higher seroconversion rates compared to intramuscular routes. For example, Polito et al. [34], in an RCT involving 92 HD patients, found that Fendrix^®^ administered intradermally (5 mcg per dose, multiple doses) led to significantly higher seroconversion than intramuscular Fendrix^®^ (20 mcg) (78% vs. 52% for intramuscular Fendrix^®^ 20 mcg; *p* < 0.05; no CI reported), although the specific rates for the IM arm in that study were lower than some other Fendrix studies [34] (no specific rates reported; concluded more effective Fendrix). The original manuscript mentioned a hypothetical 80% vs. 60% (IM) seroprotection rate from included studies, but specific primary data from the included studies supporting these exact figures for a direct comparison needs careful verification or revision to reflect findings from broader literature if not directly available from the included set. Evidences for intradermal administration, while promising, particularly for non-responders, still requires further standardization of dose and schedule in the larger trials to confirm efficacy and feasibility in routine practice **(**Table 4**).**

**Table 4.** Summary of key studies on intradermal HBV vaccination in CKD patients.

Author (Year) [Reference]	Sample Size	CKD Stage	Vaccine Type	SPR ID vs IM (%)	Statistical Significance
Fabrizi et al. (1997) [33]	50	Chronic dialysis (non-responders)	Recombinant HBV (not branded; ID: 4 mcg weekly x8; IM: 40 mcg at 0,1 mo)	52 vs 40 (at 2 mo post-series)	NS (*p* > 0.05)
Polito et al. (2011) [34]	92	Hemodialysis	Fendrix (ID: 5 mcg x4 doses; IM: 20 mcg x4 doses)	78 vs 52 (at 1 mo after the last dose)	*p* < 0.05

SPR, Seroprotection rate (anti-HBs ≥10 mIU/mL); ID, Intradermal; IM, Intramuscular; NS, Not significant; CKD, Chronic kidney disease.

Note: Studies focused on non-responders or HD patients; no data from pre-dialysis or transplant recipients in these trials.

For non-responders (anti-HBs < 10 mIU/mL post-initial series), additional doses (double-dose protocol) or switching to adjuvanted vaccines may improve seroprotection [35]. Garcia-Agudo et al. [36] reported a 97.5% (no CI reported) seroprotection rate after four doses of HepB-AS04 (Fendrix^®^) in pre-dialysis patients who had previously failed up to two cycles of four double doses of HepB-Eng (Engerix-B^®^) [36], concluding a high response with the schedule in CKD for transplantation, as the overall response was 93.8% after the full schedule (*N* = 155). Optimal strategies for non-responders remain an area requiring further study.

#### Kidney transplant recipients

Data on the efficacy of primary HBV vaccination series initiated in kidney transplant recipients (KTRs) are notably scarce. This is largely because immunosuppressive regimens, essential for preventing graft rejection, significantly impair *de novo* immune responses to vaccines [3,37]. Consequently, most clinical guidelines strongly emphasize the completion of HBV vaccination prior to transplantation [17].

When vaccination is attempted post-transplant, seroprotection rates are considerably lower than those achieved with pre-transplant vaccination. Studies suggest rates ranging from as low as 17% to potentially 50-70%, the higher end often seen if vaccination was initiated pre-transplant and completed post-transplant. The optimal timing for initiating vaccination in KTRs who were not vaccinated pre-transplant is generally recommended to be 3-6 months after transplantation, once maintenance immunosuppression levels are reached and the intensity of immunosuppression is reduced.

The included studies primarily address duration of immunity rather than primary immunogenicity **(**Table 5**)**. Sheth et al. [38] reported on the duration of immunity in pediatric dialysis patients, noting that 16-90% maintained immunity at three years, varying by age and regimen; this study did not focus on primary immunogenicity in KTRs but illustrates the challenges of maintaining immunity in immunocompromised populations [38] (*N* = 45, no CI or *p*-value for primary, concluded variable duration influenced by age and regimen). Chancharoenthana et al. [39] also studied the durability of antibody responses in KTRs who were successfully vaccinated pre-transplantation, proposing guidelines for monitoring and booster doses. Regular monitoring of anti-HBs titers (e.g., every 6–12 months) is recommended for KTRs, with booster doses considered if titers fall below 10 mIU/mL, although the most effective booster strategies in this population are not definitively established [39].

**Table 5.** Summary of studies on hepatitis B vaccination in kidney transplant recipients.

Author (Year) [Reference]	Study Design, Site	Population	Vaccine & Schedule	Sample Size (N)	SPR (%) at X Month(s) After the Last Dose (95% CI; p-value if applicable)	Duration of Response or Significant Outcomes
Sheth RD, et al. (2014) [38]	Retrospective cohort, USA	Pediatric KTRs (vaccinated pre-transplant and some progressed to transplant)	Various regimens (not specified for primary; focused on duration)	*N* = 45 (total, including dialysis)	Not reported for primary SPR in KTRs	16–90% maintained SPR at 3 years (varied by age and regimen); illustrates challenges in maintaining immunity post-transplant.
Chancharoenthana W, et al. (2019) [39]	Retrospective cohort, Thailand	Adult KTRs (vaccinated pre-transplant)	Various pre-transplant regimens (e.g. Engerix-B^®^); boosters if anti-HBs <10 mIU/mL	*N* = 257	Not reported for primary SPR; focused on durability (e.g. 95% with anti-HBsAb ≥100 IU/L at 6 months post-booster in subset)	92% persistence at 12 months if initial anti-HBs ≥100 mIU/mL vs. 44% if 10-99 mIU/mL; proposed monitoring every 6–12 months and boosters. No primary *de novo* post-transplant data.

*Note: No studies in this review focused solely on primary HBV vaccination initiated post-transplant in KTRs; most data address durability from pre-transplant vaccination or HBV management in transplant. SPR, Seroprotection rate (anti-HBs ≥10 mIU/mL); KTR, Kidney transplant recipient.

Despite the critical need for HBV protection in this group, this scoping review identified a limited number of primary studies focusing on the immunogenicity of complete HBV vaccination series initiated after kidney transplantation. This highlights a significant evidence gap, with most available data pertaining to outcomes in patients vaccinated before transplant or focusing on management of preexisting HBV infection post-transplant [40–42].

#### Pediatric CKD patients

Information on HBV vaccine immunogenicity specifically in pediatric CKD patients is also less abundant compared to the adult CKD population. Children with CKD, particularly those on dialysis or who have undergone kidney transplantation, may exhibit suboptimal responses to standard pediatric HBV vaccine schedules and often require higher antigen doses or additional doses to achieve seroprotection [38,43].

The study by Sheth et al. [38], mentioned in the context of KTRs, primarily focused on the duration of immunity in 45 pediatric dialysis patients who had been previously vaccinated, finding that immunity was maintained in 16-90% at three years, influenced by age at vaccination and the regimen used [38]. For primary immunogenicity, data are sparse. For instance, Kamath et al. [44] involving 36 children with CKD (stages II-IV and dialysis), reported a seroconversion rate of 72% (26/36, no CI reported) at 1-2 months after the last dose using a standard three-dose HBV vaccination schedule [44]. Among those who initially did not respond, only 60% (6/10, no CI reported) seroconverted at 1-2 months post-second series after a second course of vaccination. Furthermore, in this cohort, only 60% of initial responders maintained protective antibody titers at 1 year after the last dose, with a significant decline in mean antibody titers [44] **(**Table 6**)**. This underscores the challenges in both achieving and maintaining immunity in pediatric CKD patients.

**Table 6.** Summary of studies on hepatitis B vaccination in pediatric CKD patients.

Author (Year) [Reference]	Study Design, Site	Population	Vaccine & Schedule	Sample Size (N)	SPR (%) at X Month(s) After the Last Dose (95% CI; p-value if applicable)	Duration of Response or Significant Outcomes
Sheth RD, et al. (2014) [38]	Retrospective cohort, USA	Pediatric dialysis patients (some progressed to transplant)	Various regimens (not specified for primary; focused on duration)	*N* = 45	Not reported for primary SPR	16–90% maintained SPR at 3 years (influenced by age at vaccination and regimen); highlights variable durability in pediatric CKD.
Kamath N, et al. (2019) [44]	Prospective study, UAE	Pediatric CKD stages II-IV and dialysis	Standard three-dose HBV vaccine (not specified)	*N* = 36	72% (at 1-2 months after the last dose); 60% after second series for non-responders	60% of initial responders maintained protective titers at 1 year; significant titer decline observed.

*Note: Limited primary studies on HBV vaccine immunogenicity in pediatric CKD; no new data from adjuvanted vaccines or enhanced regimens specific to children. SPR, Seroprotection rate (anti-HBs ≥10 mIU/mL); CKD, Chronic kidney disease.

A most recent updated search yielded no new primary studies on HBV vaccine immunogenicity in pediatric CKD populations. This ongoing scarcity of data highlights the need for dedicated pediatric trials, potentially incorporating adjuvanted vaccines (if approved for children) or age-adjusted enhanced regimens, to address factors like growth-related immune variability and comorbidities.

Guidelines for pediatric CKD patients generally recommend adherence to age-appropriate HBV vaccination schedules but emphasize the critical need for post-vaccination serologic testing to confirm antibody response. For children who do not achieve adequate anti-HBs levels (≥10 mIU/mL), administration of additional doses or a complete repeat series, potentially with higher antigen content if available and appropriate for age, is often advised. However, this review identified few primary studies detailing the immunogenicity of various contemporary HBV vaccine formulations and intensified schedules across the spectrum of pediatric CKD stages. This represents an important area for future research to define optimal, evidence-based vaccination strategies tailored to this vulnerable young population.

#### Duration of protection

CKD patients generally exhibit not only lower initial seroconversion rates but also a more rapid decline of protective anti-HBs antibody levels and thus a shortened duration of protective immunity compared to healthy individuals [3]. In the general healthy adult population, HBV vaccine-induced immunity is robust, with over 95% achieving initial seroprotection, and this immunity can persist for at least 30 years, often for life, without the need for routine booster doses [8,9]. In contrast, the immune response in CKD patients is less durable.

Studies show that approximately 78-96% of adult dialysis patients who initially respond to vaccination maintain protective antibody levels at one year, depending on the number and type of vaccine doses given. For example, Awad et al. [27] found that 96% of hemodialysis patients who responded to a 4-dose Heplisav-B^®^ regimen maintained seroprotection at 1 year after the last dose (no CI reported). While these one-year protection rates in responders appear favorable, they must be contextualized by the often lower initial response rates in some CKD subgroups compared to healthy adults and the subsequent faster decline in antibody titers, necessitating vigilant monitoring and booster strategies not typically required in the general population [5,35,45].

The study focused on the duration of protective antibody found that maintaining anti-HBs at a level of 100 mIU/mL or more is correlated with longer persistence of protective antibody (92% remaining at 12 months after the last dose compared to 44% in the weak responder group defined as anti-HBs 10-99 mIU/mL) [39]. Several studies have demonstrated the low durability of protective immunity at three years after the last dose, ranging from 16% to 90%, depending on age group, stage of CKD, and vaccination regimen [38,45]. According to a recent survival analysis, Girndt et al. [46] reported that around 25% of participants with eGFR less than 45 mL/min/1.73 m^2^ lost seroprotection at 19 months after the last dose in the four doses of the 40 mcg HepB-Eng group and 26 months after the last dose in the three doses of the HepB-CpG group (N not specified per arm; durability similar for ≥ 10 mIU/mL, but ≥ 100 mIU/mL persisted longer in HepB-CpG, with higher geometric mean concentration over time, *p* ≤ 0.0001; no specific CI for time to loss). This study is a key source for long-term immunogenicity data comparing these two vaccine types in CKD patients, and its findings are crucial for understanding differential waning of immunity [46]. Given the shortened immunity duration in CKD patients, with a substantial proportion losing seroprotection within 1.5 to 2.5 years, annual anti-HBs monitoring and boosters for those with levels below 10 mIU/mL are suggested [5,45]. Checking anti-HBs levels 1-2 months after completion of the primary vaccination series is indeed recommended by guidelines such as KDIGO [17] and is considered standard of care in many regions for CKD patients to confirm initial seroprotection [17]. This initial check is crucial due to the recognized variability in vaccine response in this population. This is then followed by recommendations for at least annual monitoring due to the demonstrated shortened duration of immunity. Timing and frequency of booster administration require further standardization through longitudinal studies. To sum up, based on current evidence, a reasonable follow-up strategy could involve checking anti-HBs levels 1-2 months after completing the vaccination series to confirm seroprotection (anti-HBs ≥ 10 mIU/mL), followed by annual monitoring thereafter. Booster doses may be administered if levels fall below 10 mIU/mL, as studies suggest rapid declines in CKD patients [45,46]. Standardized protocols, however, require further validation through prospective studies.

#### Factors influencing vaccine seroresponse

Various factors have been identified over the years that can affect the response to hepatitis B vaccination. These factors include age, gender, diabetes, dialysis duration, nutritional status, hepatitis C virus (HCV) infection, body mass index (BMI), dialysis method, erythropoietin treatment, and genetic factors [5,47,48]. Notably, older age consistently reduces seroresponse, with studies reporting lower rates in patients over 60 years, likely due to immunosenescence. Longer dialysis duration is also associated with diminished responses, possibly reflecting cumulative immune impairment. Diabetes and HCV infection further impair seroprotection, with non-responders showing higher HCV prevalence [48,49]. The type of dialysis can have inconsistent effects, and erythropoietin treatment does not appear to significantly impact response. People carrying the HLA-DR3 allele tend to have better responses, indicating a genetic influence on immune reactions.

In addition to these factors, the use of immunosuppressive medications for underlying kidney diseases (e.g. various forms of glomerulonephritis) or for other coexisting autoimmune conditions can significantly impair vaccine seroresponse. Additionally, studies have shown that ID administration of the HBV vaccine leads to significantly higher seroprotection rates compared to the IM route, particularly in previous non-responders. Many patients with pre-dialysis CKD or even some on dialysis may be receiving such therapies. Studies in various immunocompromised populations, including those on specific immunosuppressive drugs like corticosteroids, calcineurin inhibitors, or cytotoxic agents, have consistently demonstrated reduced immunogenicity to HBV vaccines. For instance, a study on immunosuppressive treatments and risk factors in CKD patients reported that immunosuppressive therapy was associated with a significantly higher risk of non-response to hepatitis B vaccination (RR = 2.49, 95% CI: 1.26–4.96), particularly with corticosteroids and calcineurin inhibitors, which may suppress T-cell activation and antibody production essential for vaccine efficacy [50]. Similarly, one analysis evaluating immunogenicity in CKD patients noted that immunosuppressive or hormone therapy significantly attenuates protective immunity, with lower seroprotection rates observed in treated subgroups compared to untreated CKD patients [51]. These findings align with broader evidence in transplant recipients and other immunocompromised groups, where similar regimens impair responses to vaccines like HBV, suggesting a dose- and duration-dependent effect that interferes with B-cell and T-cell processes [52]. The type, dosage, and duration of immunosuppressive therapy can influence the degree of immune impairment. This highlights the clinical challenge and importance of attempting vaccination before the initiation of potent immunosuppressive treatments or, if clinically feasible, during periods of reduced immunosuppression. The variability in seroprotection rates observed even with similar vaccine regimens within CKD subpopulations may be partly attributable to the undocumented or unanalyzed impact of concurrent immunosuppressive therapy in some study cohorts. To mitigate this, future studies should systematically account for immunosuppressive use in analyses, and clinical guidelines may benefit from stratified recommendations, such as prioritizing adjuvanted vaccines for patients on these therapies.

Additionally, studies have shown that ID administration of the HBV vaccine leads to significantly higher seroprotection rates compared to the IM route, particularly in previous non-responders. A systematic review focusing on hemodialysis patients who were non-responders to IM vaccination found that ID administration resulted in significantly higher seroconversion rates. While the original manuscript cited hypothetical data of 80% vs. 60% seroprotection for ID vs. IM from studies, it is crucial to report actual data from included studies if available. If specific comparative data from the studies included in this review are lacking, then reference to systematic reviews or other primary literature supporting this should be made clear. For example, specific studies like Fabrizi et al. [33] and Polito et al. [34] explored ID Fendrix^®^ in HD patients, demonstrating good response rates, thereby supporting its use as a viable alternative for this patient group. It is important to note that ID vaccination typically requires a smaller amount of antigen to achieve an immune response, though it may involve more doses or specialized administration techniques. However, evidence from large, well-designed trials remains somewhat limited, and further research is needed to confirm efficacy, optimal dosing, and feasibility in routine clinical practice.

#### Vaccine strategies for nonresponders and current practices

Over the past year, many dialysis providers in the United States, and possibly in other countries, have started to administer the HepB-CpG vaccine (Heplisav-B^®^) to their patients based on a specific schedule [28,29]. It is anticipated that the HepB-CpG vaccination will soon replace traditional hepatitis B vaccines in dialysis units worldwide due to its potential for higher and more rapid seroprotection [19,27]. Current recommendations for individuals who previously completed a primary vaccination series but are identified as non-responders (anti-HBs <10 mIU/mL) or have experienced waning immunity include revaccination, often with a different or more potent strategy. For instance, if Heplisav-B^®^ (HepB-CpG) is used for revaccination of a prior non-responder to a conventional alum-adjuvanted vaccine, the standard 2-dose schedule (20 mcg at 0 and 1 month) is typically employed, as per CDC recommendations for adults who are unvaccinated or incompletely vaccinated [31,32]. Notably, for individuals who were previously immunized, two doses of HepB-CpG given a month apart will be effective. The standard Heplisav-B^®^ regimen for adults is two doses. If a patient is a non-responder to a previous different HBV vaccine series, they would typically receive a full new series of Heplisav-B^®^ (i.e. 2 doses). A 3-dose Heplisav-B^®^ regimen is not standard for this indication, although some studies in highly immunocompromised groups might explore alternative schedules. This point should be revised to accurately reflect standard recommendations for revaccinating non-responders with Heplisav-B^®^.

In cases of new dialysis dependence with no previous vaccination and no HepB-CpG available, it is recommended to administer four doses of HepB-Eng in double doses (some cases may require two series of HepB-Eng) [25]. When HepB-CpG is available, 2 doses given a month apart are generally expected to be efficient in achieving protective levels in treatment-naive individuals [28,31].

#### Safety

The safety profiles of conventional and adjuvanted HBV vaccines in CKD patients were generally comparable across the included studies, with no significant differences in the overall incidence or severity of adverse events between vaccine types [19,21,22,27,46]. Common adverse events were mild and transient, primarily consisting of injection site pain, myalgia, and fatigue. For example, in the trial by Janssen et al. [22] comparing Heplisav-B^®^ and Engerix-B^®^ in pre-dialysis CKD patients with type 2 diabetes (*N* = 328), solicited injection site pain was reported by 50.9% in the Heplisav-B^®^ group versus 45.5% in the Engerix-B^®^ group, and fatigue by 25.5% versus 21.8%, respectively; these differences were not statistically significant. Similarly, Awad et al. [27] reported that in 119 hemodialysis patients receiving a 4-dose schedule of Heplisav-B^®^, the most common solicited adverse reactions within 7 days of any injection were injection site pain (49.6%), fatigue (26.1%), and headache (20.2%). Serious adverse events were rare in these studies and generally not considered related to vaccination by the study investigators. A comprehensive, study-by-study breakdown of adverse event frequencies was not consistently available or reported in a comparable manner across all included studies to facilitate a pooled quantitative summary, but individual study reports generally support a favorable safety profile for the HBV vaccines used in CKD patients [18,20,29,36,53–57].

#### Heterogeneity across studies

Significant clinical and methodological heterogeneity was observed across the included studies. This included variations in CKD patient populations (e.g., mix of pre-dialysis stages with differing baseline eGFR, duration of dialysis, prevalence of comorbidities like diabetes which is a known factor for reduced vaccine response), HBV vaccine regimens (specific type of conventional or adjuvanted vaccine, antigen dose, number of doses, administration schedule, and route), definition and timing of outcome assessment (e.g., seroprotection defined as anti-HBs ≥10 mIU/mL but measured at varying intervals post-vaccination, such as 1 month, or as late as the 4th or 5th month after the final dose in multi-dose accelerated or extended schedules), and overall study designs (ranging from RCTs to observational studies with differing levels of bias control) [25,47]. For example, seroprotection rates for Engerix-B^®^ 40 mcg 4-dose regimens in pre-dialysis patients ranged broadly from 63% (95% CI: 52.4–72.9%; *N* = 67) to 100% (no CI; *N* = 19) across different studies, likely influenced by differences in baseline patient characteristics (e.g., higher eGFR in the Krairittichai study [18] vs. lower eGFR in the Kittrakulrat study [20]) and sample sizes. Similarly, for Heplisav-B^®^ in dialysis patients, reported SPRs varied from 50 to 60% in a large real-world study using a 2-dose schedule to 89.3% in an open-label study using a 4-dose schedule, potentially due to differences in the number of doses administered, study design (RCT vs. real-world effectiveness), and patient selection criteria [27,28].

Due to this substantial heterogeneity, a quantitative meta-analysis of seroprotection rates was deemed inappropriate for this scoping review. Statistical assessment of heterogeneity using metrics such as the I^2^ statistic was therefore not performed, as it is typically associated with meta-analytic approaches. The impact of this variability is a key consideration and is further discussed in the limitations section. Subgroup considerations (e.g. pre-dialysis vs. dialysis, adjuvanted vs. conventional vaccines, standard vs. accelerated schedules) are integrated into the narrative synthesis to qualitatively explore potential sources of variation and patterns in the evidence. Forest plots were not generated due to the absence of meta-analysis; however, the enhanced summary tables **(**Tables 2 and 3**)** present key outcomes, including sample sizes, confidence intervals, and p-values where available, across different CKD stages and vaccine types to facilitate a more critical qualitative comparison and to illustrate the range and variability of findings in the existing literature.

## Discussion

This scoping review has synthesized evidence on HBV vaccination strategies for patients across various stages of CKD, revealing important patterns in immunogenicity, duration of protection, influencing factors, and safety, while also highlighting significant evidence gaps. The findings underscore the complexity of achieving optimal HBV protection in this vulnerable population due to underlying immune dysregulation and the impact of renal replacement therapies [2,3].

### Summary of main findings

The primary findings of this review indicate that vaccinating CKD patients at earlier, pre-dialysis stages generally results in better immune responses compared to vaccination initiated after commencement of dialysis [16,17]. For both pre-dialysis and dialysis patients, enhanced vaccination strategies, such as four double doses of conventional alum-adjuvanted vaccines (e.g., Engerix-B^®^ 40 mcg) [25,47] or regimens using newer adjuvanted vaccines (e.g., Fendrix^®^, Heplisav-B^®^), tend to yield higher seroprotection rates than standard-dose conventional vaccine schedules typically used in the general population [19,21,30,58]. Adjuvanted vaccines appear particularly beneficial for dialysis patients, who often exhibit the poorest responses [27,28,30]. However, even with these enhanced strategies, a notable proportion of CKD patients may not achieve protective antibody levels.

The duration of vaccine-induced immunity is considerably shorter in CKD patients than in healthy individuals [8,9], with a significant decline in anti-HBs titers observed over time, often necessitating annual monitoring and booster doses [45,46]. Several factors, including older age, diabetes mellitus, longer duration of dialysis, use of immunosuppressive medications, and genetic predispositions, can negatively influence seroresponse [5,47,48]. The safety profiles of the various vaccines appear generally comparable and acceptable, with most adverse events being mild and transient. Data on optimal primary vaccination strategies initiated in kidney transplant recipients and in pediatric CKD patients remain limited, representing critical areas for further investigation [38,39].

### Strengths and limitations of the review

The strengths of this review include a comprehensive search of multiple databases, adherence to PRISMA-ScR guidelines for scoping reviews [12], and the inclusion of studies covering various CKD stages and vaccine types. Furthermore, the systematic assessment of risk of bias in included studies using standardized tools (RoB 2 for RCTs and ROBINS-I for non-randomized studies) and the application of principles from the SWiM guideline for structured narrative synthesis enhance the methodological rigor and transparency of the review.

However, several limitations must be acknowledged. First, as detailed in the Results section, significant clinical and methodological heterogeneity was present across the included studies regarding patient populations, specific interventions, outcome definitions, and study designs. This heterogeneity precluded a quantitative meta-analysis and limits the ability to make direct, definitive comparisons between all strategies. Consequently, the findings should be interpreted as a comprehensive map of the available evidence landscape rather than a precise hierarchical ranking of all possible vaccination approaches. Second, there is an underrepresentation of robust data concerning primary HBV vaccination initiated in kidney transplant recipients and in pediatric CKD patients, with only a few studies addressing these populations [38,39]. While some studies touched upon these populations, dedicated research focusing on optimal initial immunization strategies is sparse. Third, while a risk of bias assessment was conducted (Appendix A2), variations in the methodological quality of the included studies—such as some RCTs having domains with unclear risk of bias or inherent limitations in observational study designs—may influence the robustness of their individual findings and, by extension, the synthesis presented in this review. The lack of consistent, detailed statistical reporting (e.g., confidence intervals, p-values for direct comparisons) in some primary studies also hampered a more granular comparative analysis.

Fourth, long-term data on the duration of seroprotection (beyond 1–3 years for many strategies) and, more importantly, on clinical effectiveness in preventing HBV infection are limited. Fifth, the variability in seroresponse rates even within similar patient groups and vaccine regimens underscores the influence of unmeasured or inconsistently reported patient-level factors. Moreover, the underrepresentation of data on KTRs and pediatric CKD patients persists, limiting the review’s ability to provide comprehensive recommendations for these groups. Finally, the review was limited to English-language publications, which might introduce a language bias, and comprehensive cost-effectiveness analyses for many of the newer and more expensive adjuvanted vaccine strategies, particularly in diverse global economic settings, are largely unavailable in the included literature [10,23,24].

### Interpretation of findings in context of existing evidence

The findings align with KDIGO [17] and ACIP [31,32] guidelines, which advocate early vaccination in CKD, higher antigen doses, or more immunogenic adjuvanted vaccines (e.g. Fendrix^®^ and Heplisav-B^®^), with consistent evidence of improved seroprotection, particularly in dialysis patients, supporting their increasing clinical adoption where available [17,31,32]. The real-world effectiveness data, such as that from Manley et al. [28] for Heplisav-B^®^, showing somewhat lower seroprotection rates than initial RCTs, highlights the ongoing need to bridge the gap between efficacy demonstrated in controlled trial settings and actual effectiveness in broader, more heterogeneous clinical populations. This discrepancy emphasizes that factors beyond the vaccine itself, such as patient adherence, comorbidities, and variations in healthcare delivery, can significantly impact outcomes.

The challenge of managing non-responders remains a significant clinical issue. While strategies like revaccination with a different or more potent vaccine series show some success [35,36], a subset of CKD patients remains susceptible to HBV despite repeated vaccination attempts. The variability in seroprotection rates even with seemingly robust regimens, such as the 63-100% range for four double doses of Engerix-B^®^ in pre-dialysis patients [18,20], strongly suggests that individual patient factors (e.g. baseline eGFR, age, diabetes, concurrent immunosuppression) play a profound role and necessitate a more personalized approach to vaccination.

Regarding heterogeneity in the evidence (as mentioned in the Results), clinicians can generally generalize that adjuvanted vaccines (e.g. Heplisav-B^®^ or Fendrix^®^) consistently outperform conventional vaccines in achieving higher short-term seroprotection rates across pre-dialysis and dialysis stages, particularly in patients with advanced CKD or comorbidities (such as diabetes), where pooled data from RCTs and cohorts show SPRs of 89-95% versus 63-81% for enhanced conventional regimens [19,21,22,30]. This supports prioritizing adjuvanted options in these subgroups when available, as the pattern holds despite variations in study designs and populations. However, caution is advised when extrapolating to specific contexts: for instance, real-world studies often report lower SPRs (e.g. 50-60% for 2-dose Heplisav-B^®^ in dialysis [28]) due to more diverse patient characteristics (e.g. higher comorbidity burden or adherence issues) compared to controlled RCTs, suggesting that trial results may overestimate performance in heterogeneous clinical settings. Similarly, while early vaccination (pre-dialysis, eGFR >15 mL/min/1.73 m^2^) yields higher SPRs (63-100%) than in dialysis patients (50-89.3%), this difference may be confounded by unmeasured factors like dialysis duration or immunosuppression in some studies, limiting direct comparisons across stages without considering these variables. For special populations like KTRs and pediatric CKD patients **(**Tables 5 and 6**)**, generalization is particularly unreliable due to sparse data focused mainly on durability rather than primary immunogenicity, necessitating individualized strategies based on pre-transplant vaccination where possible and close monitoring. Overall, clinicians should interpret these findings cautiously in resource-limited or highly comorbid settings, tailoring choices to patient-specific factors and local vaccine availability while awaiting more standardized, subgroup-stratified trials to resolve these uncertainties.

### Implications for clinical practice

**Figure 2.**
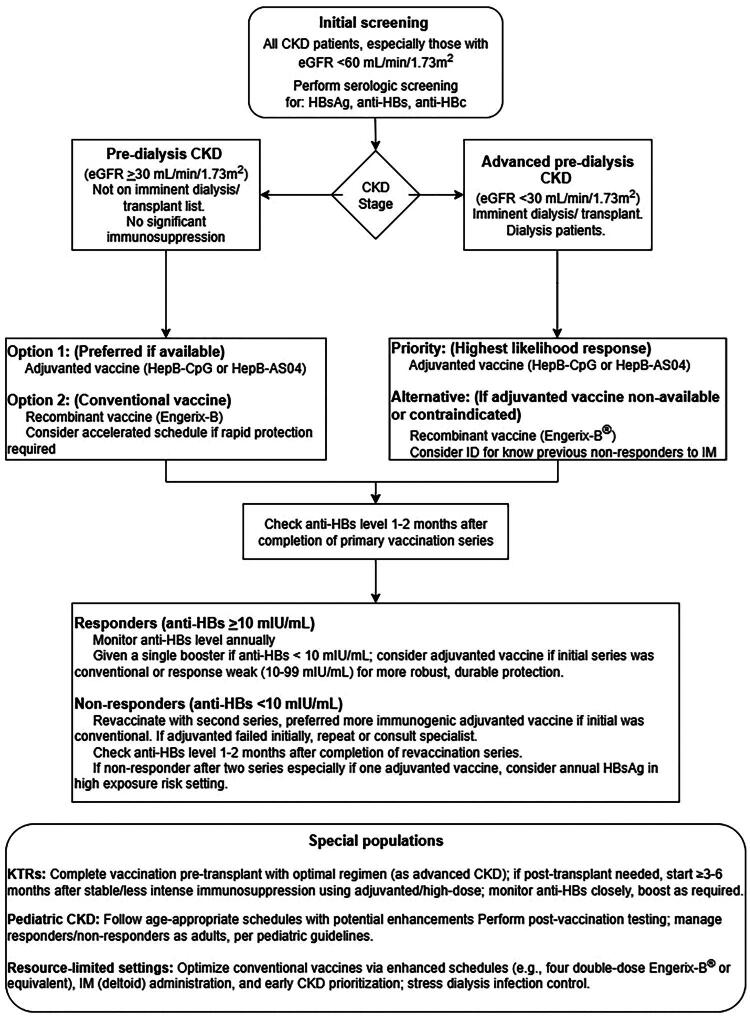
Flowchart of proposed clinical algorithm for HBV vaccination in CKD patients. This visual representation outlines a tiered approach to HBV vaccination based on CKD stage, vaccine availability, post-vaccination testing, management of responders and non-responders, special considerations for kidney transplant recipients (KTRs) and pediatric patients, and adaptations for resource-limited settings. Abbreviations: CKD, chronic kidney disease; eGFR, estimated glomerular filtration rate; ID, intradermal; IM, intramuscular; anti-HBs, hepatitis B surface antibody; HBsAg, hepatitis B surface antigen; anti-HBc, hepatitis B core antibody.

Developing a clear, universally applicable, evidence-based algorithm for HBV vaccination in CKD patients is challenging due to the existing evidence gaps and the heterogeneity of both the patient population and study findings **(**Figure 2**)**. However, based on the synthesized evidence, the following tiered approach, which considers CKD stage, vaccine availability, and patient response, can be proposed for clinical consideration. This algorithm, while evidence-informed, carries inherent uncertainties due to the limitations of the current evidence base and requires ongoing validation and adaptation based on emerging research and local resource contexts.

*Proposed Clinical Algorithm for HBV Vaccination in CKD Patients:*

1. Initial Screening (All CKD patients, especially with progressive disease or eGFR <60 mL/min/1.73 m^2^):
  - Perform serologic screening for HBsAg, anti-HBs, and total anti-HBc [17,31,32].
2. Vaccination of susceptible individuals (negative for HBsAg and anti-HBs; anti-HBc status considered for risk of reactivation if HBsAg-positive donor is contemplated for transplant):
  - Pre-dialysis CKD (e.g., eGFR ≥30 mL/min/1.73 m^2^, not on imminent dialysis/transplant list, no significant active immunosuppression):
    - Option 1 (Preferred if available/affordable): adjuvanted vaccine.
      - Heplisav-B^®^ (HepB-CpG): 20 mcg, 2 doses at 0 and 1 month [31,32].
      - Fendrix^®^ (HepB-AS04): 20 mcg, 4 doses at 0, 1, 2, and 6 months (where licensed and available) [21,30].
    - Option 2: Conventional vaccine, enhanced regimen.
      - Engerix-B^®^ (or equivalent licensed recombinant vaccine): 40 mcg (double adult dose), 4 doses at 0, 1, 2, and 6 months [19,25].
    - Consider accelerated schedules (e.g., Engerix-B^®^ 40 mcg at 0, 1, 4, and 8 weeks) if rapid protection is clinically indicated (e.g., impending need for dialysis) [20].
  - Advanced pre-dialysis CKD (e.g., eGFR < 30 mL/min/1.73 m^2^ or imminent dialysis/transplantation)/dialysis patients (hemodialysis or peritoneal dialysis):
    - Priority (highest likelihood of response): adjuvanted vaccine.
      - Heplisav-B^®^: 20 mcg, 2 doses at 0 and 1 month. Some studies in HD have used 3 or 4 doses; however, the 2-dose schedule is standard for adults and has shown efficacy [27–29,31,32].
      - Fendrix^®^: 20 mcg, 4 doses at 0, 1, 2, and 6 months (where licensed and available) [28,30].
    - Alternative (if adjuvanted vaccines are unavailable, contraindicated, or unaffordable): conventional vaccine, enhanced regimen.
      - Engerix-B^®^ (or equivalent): 40 mcg, 4 doses at 0, 1, 2, and 6 months [25].
      - Consider intradermal administration of conventional vaccine for known previous non-responders to IM conventional vaccine, if expertise and appropriate formulation are available [34].
3. Post-vaccination serologic testing (all CKD patients):
  - Check anti-HBs level 1-2 months after completion of the primary vaccination series [17,31,32].
4. Management based on post-vaccination anti-HBs level:
  - Responder (anti-HBs ≥ 10 mIU/mL):
    - Monitor anti-HBs annually [5,45,46].
    - Administer a single booster dose if the anti-HBs level falls below 10 mIU/mL. Consider using an adjuvanted vaccine for the booster if the initial series was with a conventional vaccine or if the initial response was weak (e.g., 10-99 mIU/mL) to potentially achieve a more robust and durable response [35,39].
  - Non-responder (anti-HBs < 10 mIU/mL):
    - Revaccinate with a complete second series, preferably using a different (more immunogenic) strategy if the first series was with a conventional vaccine (e.g., switch to an adjuvanted vaccine like the Heplisav-B^®^ 2-dose series) [31,32,36]. If an adjuvanted vaccine was used initially and failed, options are limited but may include repeating the same adjuvanted vaccine or consulting specialist advice.
    - Retest anti-HBs 1-2 months after completion of the revaccination series.
    - If still a non-responder after two full vaccination series (especially if one involved an adjuvanted vaccine), the patient is considered a persistent non-responder. Counsel on HBV prevention measures and the continued risk of infection. Consider annual HBsAg testing for early detection of infection, particularly in high-prevalence settings or if an exposure risk exists.
5. Special populations:
  - Kidney transplant recipients: Should complete vaccination pre-transplant using an optimal regimen (as above for advanced CKD) [17]. If vaccination is required post-transplant, initiate the vaccination ≥ 3-6 months after transplantation when immunosuppression is stable and less intense. Use adjuvanted or high-dose regimens. Monitor anti-HBs levels closely and provide boosters as needed [39].
  - Pediatric CKD patients: Follow age-appropriate schedules, but anticipate the need for enhanced strategies (e.g., higher doses if licensed for age, or adjuvanted vaccines if available and approved for pediatric use in this context – current adjuvanted vaccines like Heplisav-B^®^ and Fendrix^®^ are primarily for adults). Conduct post-vaccination serologic testing and manage responders/non-responders similarly to adults, with consideration for pediatric-specific guidelines [38].
6. Considerations for resource-limited settings
  - In resource-limited settings where newer adjuvanted vaccines may be prohibitively expensive or unavailable, optimizing the use of conventional recombinant vaccines is paramount. This includes strict adherence to enhanced schedules (e.g., four double doses of Engerix-B^®^ or equivalent), ensuring intramuscular (deltoid) administration, and prioritizing vaccination at the earliest possible stage of CKD [10,25].
  - Emphasis on infection control measures in dialysis units also remains critical [2].

### Implications for future research

This review identifies several key areas for future research. There is a pressing need for large-scale, well-designed RCTs that directly compare newer adjuvanted vaccines (e.g., Heplisav-B^®^ vs. Fendrix^®^, where both might be available options in some regions, or future adjuvanted candidates) and also compare these adjuvanted vaccines against optimized conventional regimens (e.g., four double doses) across different, well-defined CKD stages. Such trials should include long-term follow-up (ideally 3–5 years or more) to assess not only initial seroprotection but also the duration of immunity and, crucially, clinical effectiveness in preventing HBV infection and related complications.

Further research is essential to determine the optimal strategies for managing non-responders, including the comparative efficacy of different revaccination approaches. Dedicated studies are critically needed for pediatric CKD patients and kidney transplant recipients to establish the most effective primary vaccination regimens, as current data are insufficient **(**Tables 5 and 6**).** The impact of specific types and intensities of immunosuppressive therapies on HBV vaccine response in CKD patients warrants more detailed investigation. Additionally, further validation of intradermal vaccination routes, including optimal antigen doses and schedules for this method, could provide valuable alternatives, especially for non-responders. Finally, robust cost-effectiveness studies are required to guide the implementation of different vaccination strategies, particularly comparing adjuvanted vaccines with enhanced conventional regimens, in various healthcare systems and resource settings. The findings from such research will be instrumental in refining clinical guidelines and ensuring that HBV vaccination strategies for CKD patients are both maximally effective and equitably accessible.

## Conclusion

This scoping review highlights the critical importance of effective hepatitis B vaccination for patients with CKD, a population at heightened risk of HBV infection and its sequelae due to compromised immune function and frequent healthcare exposures [2,3]. The evidence synthesized supports early vaccination, ideally in the pre-dialysis stages of CKD, to achieve better immune responses [16,17]. Enhanced vaccination strategies, including regimens with four double doses of conventional recombinant vaccines or the use of newer adjuvanted vaccines like HepB-AS04 (Fendrix^®^) and HepB-CpG (Heplisav-B^®^), generally demonstrate superior seroprotection rates compared to standard approaches, particularly in patients with advanced CKD and those on dialysis [19,21,27,30].

However, significant heterogeneity exists in the reported outcomes, and the duration of protection is often limited, necessitating individualized approaches that include post-vaccination serologic testing and tailored booster strategies. Critical evidence gaps remain, especially concerning optimal primary vaccination in kidney transplant recipients and pediatric CKD patients, with no new data identified, highlighting the urgency for dedicated research in these areas. The review’s findings emphasize the need for high-quality, large-scale randomized controlled trials to address these uncertainties, compare the long-term benefits of different vaccine strategies head-to-head, and standardize protocols for vaccination, monitoring, and management of non-responders. Ultimately, while current strategies offer improved means of protection, continued research and diligent clinical application are paramount to minimizing the burden of HBV infection in the vulnerable CKD population.

## Supplementary Material

Appendix A.docx

Appendix B.xlsx

## Data Availability

The datasets used and generated in this study are included in this published article and its supplementary information files.

## References

[1] World Health Organization. *Global hepatitis report 2024: action for access in low- and middle-income countries*; 2024.

[2] Khalesi Z, Razizadeh MH, Javadi M, et al. Global epidemiology of hbv infection among hemodialysis patients: a systematic review and meta-analysis. Microb Pathog. 2023;179:106080. doi:10.1016/j.micpath.2023.106080. 36948364

[3] Kato S, Chmielewski M, Honda H, et al. Aspects of immune dysfunction in end-stage renal disease. Clin J Am Soc Nephrol. 2008;3(5):1526–1533. doi:10.2215/CJN.00950208. 18701615 PMC4571158

[4] Grzegorzewska AE. Hepatitis b vaccination in chronic kidney disease: review of evidence in non-dialyzed patients. Hepat Mon. 2012;12(11):e7359. doi:10.5812/hepatmon.7359. 23326280 PMC3546461

[5] Udomkarnjananun S, Takkavatakarn K, Praditpornsilpa K, et al. Hepatitis b virus vaccine immune response and mortality in dialysis patients: a meta-analysis. J Nephrol. 2020;33(2):343–354. doi:10.1007/s40620-019-00668-1. 31701375

[6] Lin SY, Liu JH, Wang SM, et al. Association of response to hepatitis b vaccination and survival in dialysis patients. BMC Nephrol. 2012;13(1):97. doi:10.1186/1471-2369-13-97. 22935561 PMC3471045

[7] Sam R, Rankin L, Ulasi I, et al. Vaccination for patients receiving dialysis. Kidney Med. 2024;6(3):100775. doi:10.1016/j.xkme.2023.100775. 38435066 PMC10906410

[8] Cocchio S, Baldo V, Volpin A, et al. Persistence of anti-hbs after up to 30 years in health care workers vaccinated against hepatitis b virus. Vaccines (Basel). 2021;9(4):323. 10.3390/vaccines9040323 PMC8067181 33915763

[9] Dini G, Toletone A, Barberis I, et al. Persistence of protective anti-hbs antibody levels and anamnestic response to hbv booster vaccination: a cross-sectional study among healthcare students 20 years following the universal immunization campaign in Italy. Hum Vaccin Immunother. 2017;13(2):440–444. doi:10.1080/21645515.2017.1264788. 27925503 PMC5328216

[10] Hirst A, Hyer RN, Janssen RS. Comparative cost-effectiveness of a 2-dose versus 3-dose vaccine for hepatitis b prevention in selected adult populations. Vaccine. 2021;39(33):4733–4741. doi:10.1016/j.vaccine.2021.05.020. 34030898

[11] Aromataris E LC, Porritt K, Pilla B, Jordan Z, editors. *Jbi manual for evidence synthesis.* https://synthesismanual.jbi.global. doi:10.46658/JBIMES-24-01.(25 November 2024, date last accessed).

[12] Tricco AC, Lillie E, Zarin W, et al. Prisma extension for scoping reviews (prisma-scr): checklist and explanation. Ann Intern Med. 2018;169(7):467–473. doi:10.7326/M18-0850. 30178033

[13] Sterne JAC, Savović J, Page MJ, et al. Rob 2: a revised tool for assessing risk of bias in randomised trials. BMJ. 2019;366:l4898. doi:10.1136/bmj.l4898. 31462531

[14] Sterne JA, Hernán MA, Reeves BC, et al. Robins-i: a tool for assessing risk of bias in non-randomised studies of interventions. BMJ. 2016;355:i4919. doi:10.1136/bmj.i4919. 27733354 PMC5062054

[15] Campbell M, McKenzie JE, Sowden A, et al. Synthesis without meta-analysis (swim) in systematic reviews: reporting guideline. BMJ. 2020;368:l6890. doi:10.1136/bmj.l6890. 31948937 PMC7190266

[16] Hashemi B, Mahdavi-Mazdeh M, Abbasi M, et al. Efficacy of hbv vaccination in various stages of chronic kidney disease: is earlier better? Hepat Mon. 2011;11(10):816–820. doi:10.5812/kowsar.1735143X.1771. 22224080 PMC3234577

[17] Drüeke TB, Parfrey PS. Summary of the KDIGO guideline on anemia and comment: reading between the (guide)line(s). Kidney Int. 2012;82(9):952–960. doi:10.1038/ki.2012.270. 22854645

[18] Krairittichai U, Sethakarun S. A randomized controlled trial of seroconversion after 20 mg versus 40 mg intramuscular hepatitis b virus vaccination in patients with chronic kidney disease stage 3. J Med Assoc Thai. 2017;100 Suppl 1(Suppl 1):S1–S7. 29926711

[19] Janssen RS, Mangoo-Karim R, Pergola PE, et al. Immunogenicity and safety of an investigational hepatitis b vaccine with a toll-like receptor 9 agonist adjuvant (hbsag-1018) compared with a licensed hepatitis b vaccine in patients with chronic kidney disease. Vaccine. 2013;31(46):5306–5313. doi:10.1016/j.vaccine.2013.05.067. 23727422

[20] Kittrakulrat J, Tiankanon K, Kerr SJ, et al. A randomized controlled study of efficacy and safety of accelerated versus standard hepatitis b vaccination in patients with advanced ckd. Kidney Int Rep. 2024;9(4):853–862. doi:10.1016/j.ekir.2024.01.014. 38770057 PMC11103956

[21] Fabrizi F, Cerutti R, Nardelli L, et al. Hbv vaccination with fendrix is effective and safe in pre-dialysis ckd population. Clin Res Hepatol Gastroenterol. 2020;44(1):49–56. doi:10.1016/j.clinre.2019.06.010. 31327620

[22] Janssen JM, Heyward WL, Martin JT, et al. Immunogenicity and safety of an investigational hepatitis b vaccine with a toll-like receptor 9 agonist adjuvant (hbsag-1018) compared with a licensed hepatitis b vaccine in patients with chronic kidney disease and type 2 diabetes mellitus. Vaccine. 2015;33(7):833–837. doi:10.1016/j.vaccine.2014.12.060. 25576215

[23] Kuan RK, Janssen R, Heyward W, et al. Cost-effectiveness of hepatitis b vaccination using heplisav^™^ in selected adult populations compared to engerix-b^®^ vaccine. Vaccine. 2013;31(37):4024–4032. doi:10.1016/j.vaccine.2013.05.014. 23707166

[24] Vilajeliu A, Sequera VG, García-Basteiro AL, et al. Immunogenicity and immunization costs of adjuvanted versus non-adjuvanted hepatitis b vaccine in chronic kidney disease patients. Hum Vaccin Immunother. 2016;12(9):2317–2321. doi:10.1080/21645515.2016.1168955. 27105182 PMC5027724

[25] Mulley WR, Le ST, Ives KE. Primary seroresponses to double-dose compared with standard-dose hepatitis b vaccination in patients with chronic kidney disease: a systematic review and meta-analysis. Nephrol Dial Transplant. 2017;32(1):136–143. doi:10.1093/ndt/gfv443. 26763670

[26] Lacson E, Teng M, Ong J, et al. Antibody response to engerix-b and recombivax-hb hepatitis b vaccination in end-stage renal disease. Hemodial Int. 2005;9(4):367–375. doi:10.1111/j.1492-7535.2005.01155.x. 16219057

[27] Awad AM, Ntoso A, Connaire JJ, et al. An open-label, single-arm study evaluating the immunogenicity and safety of the hepatitis b vaccine hepb-cpg (heplisav-b^®^) in adults receiving hemodialysis. Vaccine. 2021;39(25):3346–3352. doi:10.1016/j.vaccine.2021.05.003. 34001345

[28] Manley HJ, Aweh G, Frament J, et al. A real world comparison of hepb (engerix-b^®^) and hepb-cpg (heplisav-b^®^) vaccine seroprotection in patients receiving maintenance dialysis. Nephrol Dial Transplant. 2023;38(2):447–454. doi:10.1093/ndt/gfac039. 35150277

[29] Walsh C, McDaniel K, Lindsey L, et al. Seroconversion following heplisav-b, hepatitis b vaccine (recombinant), adjuvanted, in patients with end-stage renal disease at an urban safety net hospital. Am J Health Syst Pharm. 2023;80(Suppl 4):S130–S134. doi:10.1093/ajhp/zxad022. 36681904

[30] Tong NK, Beran J, Kee SA, et al. Immunogenicity and safety of an adjuvanted hepatitis b vaccine in pre-hemodialysis and hemodialysis patients. Kidney Int. 2005;68(5):2298–2303. doi:10.1111/j.1523-1755.2005.00689.x. 16221232

[31] Schillie S, Vellozzi C, Reingold A, et al. Prevention of hepatitis b virus infection in the united states: recommendations of the advisory committee on immunization practices. MMWR Recomm Rep. 2018;67(1):1–31. doi:10.15585/mmwr.rr6701a1. PMC5837403 29939980

[32] Wodi AP, Issa AN, Moser CA, et al. Advisory committee on immunization practices recommended immunization schedule for adults aged 19 years or older - united states, 2025. MMWR Morb Mortal Wkly Rep. 2025;74(2):30–33. doi:10.15585/mmwr.mm7402a3. 39820474 PMC11737654

[33] Fabrizi F, Andrulli S, Bacchini G, et al. Intradermal versus intramuscular hepatitis b re-vaccination in non-responsive chronic dialysis patients: a prospective randomized study with cost-effectiveness evaluation. Nephrol Dial Transplant. 1997;12(6):1204–1211. doi:10.1093/ndt/12.6.1204. 9198052

[34] Polito P, Di Lullo L, Iannacci GR, et al. [seroconversion and immune response after anti-hbv vaccination in patients on chronic hemodialysis: comparison of two vaccines]. G Ital Nefrol. 2011;28(5):525–530. 22028266

[35] Chaves SS, Daniels D, Cooper BW, et al. Immunogenicity of hepatitis b vaccine among hemodialysis patients: effect of revaccination of non-responders and duration of protection. Vaccine. 2011;29(52):9618–9623. doi:10.1016/j.vaccine.2011.10.057. 22044739

[36] García-Agudo R, Aoufi Rabih S, Araque Torres P, et al. Efficacy of a hepatitis b vaccination schedule with two cycles of four double doses of conventional vaccine and four doses of adjuvanted vaccine in chronic kidney disease patients evaluated for renal transplantation. Transplant Proc. 2012;44(9):2532–2534. doi:10.1016/j.transproceed.2012.09.046. 23146445

[37] Danziger-Isakov L, Kumar D, AST Infectious Diseases Community of Practice. Vaccination in solid organ transplantation. Am J Transplant. 2013;13 Suppl 4:311–317. doi:10.1111/ajt.12122. 23465023

[38] Sheth RD, Peskin MF, Du XL. The duration of hepatitis b vaccine immunity in pediatric dialysis patients. Pediatr Nephrol. 2014;29(10):2029–2037. doi:10.1007/s00467-014-2822-7. 24839216

[39] Chancharoenthana W, Leelahavanichkul A, Udomkarnjananun S, et al. Durability of antibody response against the hepatitis b virus in kidney transplant recipients: a proposed immunization guideline from a 3-year follow-up clinical study. Open Forum Infect Dis. 2019;6(1):ofy342. doi:10.1093/ofid/ofy342. 30697573 PMC6330517

[40] Chancharoenthana W, Townamchai N, Pongpirul K, et al. The outcomes of kidney transplantation in hepatitis b surface antigen (hbsag)-negative recipients receiving graft from hbsag-positive donors: a retrospective, propensity score-matched study. Am J Transplant. 2014;14(12):2814–2820. doi:10.1111/ajt.12921. 25395260

[41] Wang XD, Liu JP, Song TR, et al. Kidney transplantation from hepatitis b surface antigen (hbsag)-positive living donors to hbsag-negative recipients: clinical outcomes at a high-volume center in china. Clin Infect Dis. 2021;72(6):1016–1023. doi:10.1093/cid/ciaa178. 32100025

[42] Thongprayoon C, Chokesuwattanaskul R, Bathini T, et al. Epidemiology and prognostic importance of atrial fibrillation in kidney transplant recipients: a meta-analysis. J Clin Med. 2018;7(10):370. 30347721 10.3390/jcm7100370 PMC6210475

[43] Neu AM. Immunizations in children with chronic kidney disease. Pediatr Nephrol. 2012;27(8):1257–1263. doi:10.1007/s00467-011-2042-3. 22048175 PMC3382633

[44] Kamath N, Vasudevan A, Iyengar A. Seroconversion following hepatitis b vaccination in children with chronic kidney disease. Saudi J Kidney Dis Transpl. 2019;30(2):334–338. doi:10.4103/1319-2442.256840. 31031369

[45] Yao T, Shao Z, Wu L, et al. Long-term persistent immunogenicity after successful standard and triple-dosed hepatitis b vaccine in hemodialysis patients: a 3-year follow-up study in china. Vaccine. 2021;39(18):2537–2544. doi:10.1016/j.vaccine.2021.03.074. 33814231

[46] Girndt M, Houser P, Manllo-Karim R, et al. Long-term immunogenicity and safety of the hepatitis b vaccine hepb-cpg (heplisav-b) compared with hepb-eng (engerix-b) in adults with chronic kidney disease. Vaccine. 2023;41(20):3224–3232. doi:10.1016/j.vaccine.2023.04.028. 37085451

[47] Fabrizi F, Donato FM, Messa P. Efficacy and safety of reinforced versus standard vaccine schedule towards hepatitis b in chronic kidney disease: a systematic review and meta-analysis. Hepat Mon. 2017;17(7):e44179. doi:10.5812/hepatmon.44179.

[48] Ferreira TMB, Guimarães TGS, Fontenele AMM, et al. Does infection by the hepatitis c virus decrease the response of immunization against the hepatitis b virus in individuals undergoing dialysis? J Bras Nefrol. 2017;39(2):141–145. doi:10.5935/0101-2800.20170020. 28489180

[49] Kdigo 2018 clinical practice guideline for the prevention, diagnosis, evaluation, and treatment of hepatitis c in chronic kidney disease. Kidney Int Suppl (2011). 2018;8:91–165. 30675443 10.1016/j.kisu.2018.06.001 PMC6336217

[50] Padilla-Matas R, Salguero-Cano V, Soler-Iborte E, et al. Immunosuppressive treatments and risk factors associated with non-response to hepatitis b vaccination: a cohort study. Vaccines (Basel). 2025;13(2):13. doi:10.3390/vaccines13020184. PMC11861145 40006731

[51] Gao L, Cui X, Mo X, et al. Immunogenicity of short-course, high-dose hepatitis b vaccination in patients with chronic kidney disease - shanxi province, china, 2019-2020. China CDC Wkly. 2024;6(50):1331–1336. doi:10.46234/ccdcw2024.264. 39734783 PMC11673178

[52] Meyer KC, Decker C, Baughman R. Toxicity and monitoring of immunosuppressive therapy used in systemic autoimmune diseases. Clin Chest Med. 2010;31(3):565–588. doi:10.1016/j.ccm.2010.05.006. 20692548

[53] Fabrizi F, Tarantino A, Castelnovo C, et al. Recombinant hepatitis b vaccine adjuvanted with as04 in dialysis patients: a prospective cohort study. Kidney Blood Press Res. 2015;40(6):584–592. doi:10.1159/000368534. 26566033

[54] McNulty CA, Bowen JK, Williams AJ. Hepatitis b vaccination in predialysis chronic renal failure patients a comparison of two vaccination schedules. Vaccine. 2005;23(32):4142–4147. doi:10.1016/j.vaccine.2005.03.020. 15913854

[55] Hettenbaugh J, Mullane R, Gillispie G, et al. Hepatitis b vaccination in advanced chronic kidney disease: a quality improvement project at a veteran affairs chronic kidney disease clinic. Infect Dis Rep. 2021;13(4):1036–1042. doi:10.3390/idr13040094. 34940404 PMC8701395

[56] Feng Y, Yao T, Han Y, et al. Immunogenicity and safety of a high-dose and prolonged-schedule hepatitis b vaccine among chronic kidney disease patients: a randomized, parallel-controlled trial. Expert Rev Vaccines. 2021;20(6):743–751. doi:10.1080/14760584.2021.1915777. 34058948

[57] Chow KM, Lo SH, Szeto CC, et al. Extra-high dose hepatitis b vaccination for peritoneal dialysis patients: a randomised controlled trial. Hong Kong Med J. 2012;18 Suppl 6(Suppl 6):41–43. 23249854

[58] Fabrizi F, Dixit V, Messa P, et al. Hepatitis b virus vaccine in chronic kidney disease: improved immunogenicity by adjuvants? A meta-analysis of randomized trials. Vaccine. 2012;30(13):2295–2300. doi:10.1016/j.vaccine.2012.01.064. 22285268

